# Impact of an Evidence-Based Prioritization System and Electronic Consultation in Early Diagnosis of Colorectal Cancer

**DOI:** 10.3390/healthcare12020194

**Published:** 2024-01-13

**Authors:** Francisco Valverde-López, Marta Librero-Jiménez, Raúl Fernández-García, Teresa Vezza, Clara Heredia-Carrasco, Mercedes López de Hierro Ruiz, Julio Galvez, Rita Jiménez-Rosales, Eduardo Redondo-Cerezo

**Affiliations:** 1 Servicio de Aparato Digestivo, Hospital Universitario Virgen de las Nieves, Avenida de las Fuerzas Armadas, 2, 18014 Granada, Spain; fcoval89@ugr.es (F.V.-L.); marta.librero.sspa@juntadeandalucia.es (M.L.-J.); raul.fernandez.garcia.sspa@juntadeandalucia.es (R.F.-G.); teresavezza@hotmail.it (T.V.); clara.heredia.sspa@juntadeandalucia.es (C.H.-C.); mariam.lopezhierro.sspa@juntadeandalucia.es (M.L.d.H.R.); eredondoc@ugr.es (E.R.-C.); 2 Instituto de Investigación Biosanitaria ibs.GRANADA, 18012 Granada, Spain; jgalvez@ugr.es; 3 Department of Medicine, Universidad de Granada, 18016 Granada, Spain; 4 Servicio de Aparato Digestivo, Hospital de Motril, 18600 Granada, Spain

**Keywords:** colorectal cancer, patient outcomes, electronic consultation, healthcare communication, telemedicine

## Abstract

(1) Background: Colorectal cancer (CRC) is one of the most common causes of cancer. Timely diagnosis is critical, with even minor delays impacting prognosis. Primary care providers face obstacles in accessing specialist care. This study investigates the impact of implementing an electronic consultation (eConsult) system combined with a specific prioritization system on CRC diagnosis delay and tumor staging. (2) Methods: The study analyzes 245 CRC patients from November 2019 to February 2022, comparing those referred before and after the eConsult system’s implementation during the COVID-19 pandemic. Data on referral reasons, pathways, diagnosis delays, and staging were collected. Multivariate analysis aimed to identify independent risk factors for advanced staging at diagnosis. (3) Results: The eConsult system significantly reduced CRC diagnosis delay from 68 to 26 days. The majority of patients referred via eConsult presented with symptoms. Despite expedited diagnoses, no discernible difference in CRC staging emerged between eConsult and traditional referrals. Notably, patients from screening programs or with a positive fecal immunochemical test (FIT) experienced earlier-stage diagnoses. A positive FIT without symptoms and being a never-smoker emerged as protective factors against advanced-stage CRC. (4) Conclusions: This study highlights eConsult’s role in reducing CRC diagnosis delay, improving diagnostic efficiency and prioritizing urgent cases, emphasizing FIT effectiveness.

## 1. Introduction

Colorectal cancer (CRC) stands as the third most prevalent cause of cancer globally and claims the unfortunate rank of being the second most lethal cancer, contributing to 9.4% of cancer-related deaths [1]. Moreover, numerous studies underscore the significant repercussions of even a few months’ delay in diagnosis, manifesting in heightened costs and diminished survival rates [2,3]. Primary care providers encounter different barriers to accessing specialist care, often struggling to convey their clinical impressions or the urgency of early intervention [4]. The Spanish guidelines strongly advocate for swift and efficient communication channels between general practitioners (GPs) and endoscopic units to expedite the early diagnosis of colorectal cancer [5]. This predicament has been exacerbated by the SARS-CoV-2 pandemic in 2019, which prompted a reallocation of medical and human resources to the COVID-19 response, leading to the deprioritization of critical medical issues such as CRC diagnosis [3,6].

Against this backdrop, the necessity to minimize the risk of exposure has spurred the adoption of novel communication methods with patients and among healthcare providers, notably through electronic consultation (eConsult) and telemedicine [7,8]. These modalities persist as routine tools even beyond the pandemic’s conclusion. The efficacy of eConsults or tele-triage programs facilitating swift specialist consultations has been well-documented in various medical disciplines, including Dermatology or Radiology [9,10,11], and is poised to extend to other domains of medicine. However, their impact on the early diagnosis of gastrointestinal neoplasms has been scarcely assessed [12] even though certain conditions, such as colorectal cancer (CCR), gastric cancer, or esophageal cancer, could benefit from prompt consultations with a general practitioner (GP) that may lead to a diagnostic endoscopic procedure. To accurately and uniformly triage patients for an endoscopic procedure, a prioritizing system is essential. In this context, the Spanish Society of Endoscopy Guidelines for Patient Prioritization developed a system for prioritizing endoscopic referrals to reduce the diagnosis delay of critical gastrointestinal diseases during the pandemic [13]. This system can also be utilized beyond the pandemic setting to prioritize urgent cases, such as those with a high suspicion of gastrointestinal neoplasms or inflammatory bowel diseases. Hence, the integration of eConsults, coupled with a dedicated prioritization system based on symptoms and details, endorsed by primary care providers to assess the urgency of an early colonoscopy, holds promise for reducing diagnosis delays and improving oncological outcomes. The primary objective of this study is to evaluate the delay in CRC diagnosis by comparing referrals to our Gastroenterology department before and after the implementation of eConsults combined with a specific prioritization system. Additionally, we aim to identify the factors influencing tumor stage at the time of diagnosis.

## 2. Materials and Methods

### 2.1. Study Design and Population

We conducted a retrospective study based on prospective registry analysis on consecutive patients diagnosed with colorectal cancer (CRC) in our Endoscopy Unit between November 2019 and February 2022, aiming to assess diagnosis delay and tumor stage at diagnosis. After the diagnosis in the endoscopy suite, patients were sent to our CRC outpatient clinic for extension study and follow-up.

The inclusion criteria comprised patients aged 18 years and above, diagnosed with colorectal cancer (CRC) through colonoscopy. Staging for colon cancer was based on a computed tomography (CT) scan, while for rectal cancer, staging included both CT scans and pelvic magnetic resonance imaging. Patients needed to possess a comprehensive diagnosis and extension study, and willingly participate in the registry. Exclusion criteria were applied to patients failing to meet the inclusion criteria, particularly those lost to follow-up or opting for management in another healthcare facility. Importantly, no other conditions such as severe comorbidities or previous malignancies led to exclusion from the study as long as they met the inclusion criteria. After extension study and treatment decisions made by a multidisciplinary committee, every patient enrolled in the registry received ongoing monitoring by our oncology department until either their demise or definitive discharge from our follow-up protocol.

### 2.2. The eConsult System

Following the onset of the COVID-19 pandemic, we implemented an exclusive and streamlined patient referral system known as the eConsult system. This electronic program was purposefully designed as a direct and almost instantaneous communication tool connecting our extensive network of almost 300 family physicians (GPs) with the Gastroenterology department. Functioning as a consultation channel, the system facilitates the resolution of specific queries concerning patients, enabling their evaluation and prioritization. The system is used for direct consultations, from the normal activity of the GPs, as well as for referrals to our outpatient clinic for any clinical situation that needs our assessment, and as the main pathway to send patients to our endoscopy unit from primary care. After the pandemic, we established this system as the only way to refer patients from primary care, keeping open the Emergency Unit referrals and other specialties referrals, which were applicable for the minority of patients.

Patients were triaged for either direct endoscopy, aligning with the Spanish Society of Endoscopy Guidelines for Patient Prioritization [13], which delineates up to five priority levels. Priority 1 refers to patients with a high risk of organic and relevant disease, such as CRC, and prompts endoscopic study in less than two weeks. Priority 2 refers to the need for a colonoscopy in less than four weeks, with the rest of patients being considered less urgent, sometimes being a review of previous disease, so that longer delays are negligible. This guideline was used to establish a local referral protocol, adapted to the circumstances of our primary care. Alternatively, patients may be recommended for an outpatient gastrointestinal (GI) visit. Frequently, Gastroenterologists provide direct treatment recommendations based on the GP’s clinical history, avoiding direct clinical contact with Gastroenterologists, and so selecting the patients who must be treated as a priority, alleviating pressure on our outpatient clinic and on our endoscopy unit.

**Figure 1:**
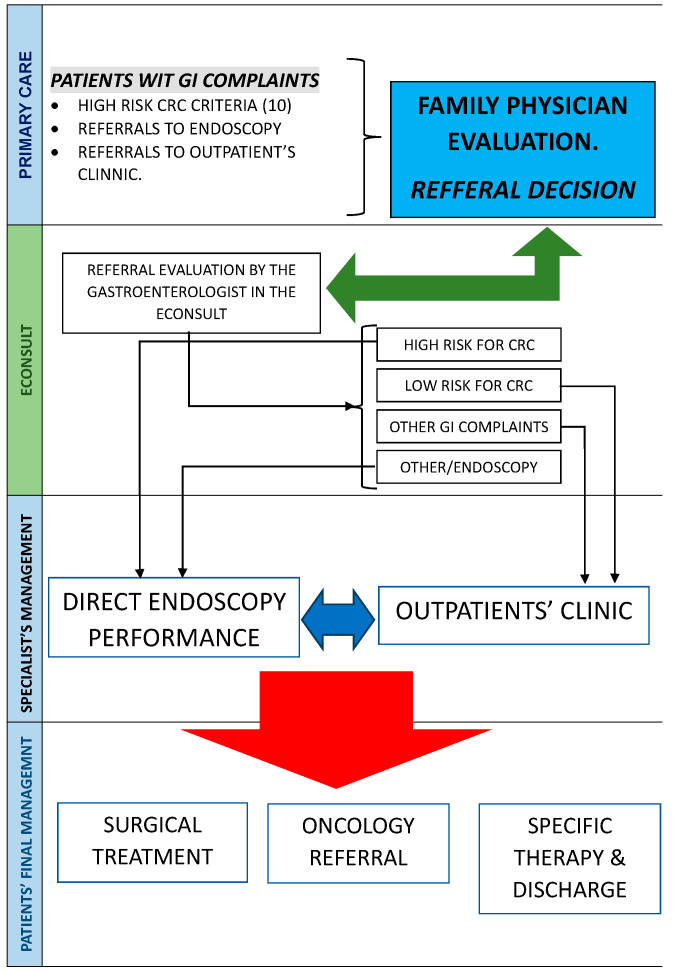
eConsult system as the main instrument to manage patients’ pathway from primary care to specific management.

The system has been fine-tuned to minimize unnecessary visits to the GI department, prioritize patients with a more severe clinical presentation, and establish a seamless and constructive collaboration with GPs, who are regarded as the keystone of patient management within our public healthcare service (Figure 1).

### 2.3. Data Source and Analysis

Our follow-up program focused on gathering data including age, sex, comorbidities, with a primary focus on diabetes, current or past smoking habits, and obesity. Referral motives to our unit were meticulously recorded, distinguishing between asymptomatic patients with a positive fecal immunochemical test (FIT) within or outside the CRC program, those with a family history of CRC, individuals presenting with anemia, and those exhibiting typical symptoms such as hematochezia, changing bowel habits, abdominal pain, constitutional symptoms, or a combination of these symptoms.

Furthermore, the study scrutinized the patients’ referral pathways. From the summer of 2020 onward, referrals originated from the electronic consultation (eConsult) program, inpatients, and patients from our emergency room, Gastroenterology outpatient clinic, and other specialty outpatient clinics (e.g., Surgery or Internal Medicine). Before this date, referrals were also directly made by general practitioners (GPs).

Finally, the registry captured the year and month of diagnosis, diagnostic delay from the initial contact with the GP, and tumor location and staging according to the 8th edition of the Tumor, Node, Metastasis (TNM) staging system established by the combined American Joint Committee on Cancer (AJCC) and the Union for International Cancer Control (UICC).

### 2.4. Statistical Analysis

The statistical analysis was conducted utilizing IBM SPSS Statistics 21.0. All tests were two-sided, and a significance level of *p* < 0.05 was deemed statistically significant. The patient data were initially compared between the periods before and after the full implementation of eConsult. Subsequently, comparisons were made between patients referred for a positive FIT test or in the context of CRC screening and those referred for other indications. Finally, patient characteristics were compared based on tumoral stage (0–II vs. III–IV). Group comparisons were executed using the chi-square test for categorical variables and Student’s *t*-test for normally distributed continuous variables. In instances where the variables did not adhere to a normal distribution, the non-parametric Wilcoxon rank-sum test was applied.

For a more comprehensive examination of independent risk factors and advanced staging at diagnosis, multivariate analysis was undertaken through a stepwise logistic regression analysis. This approach allowed for the identification of significant variables that contribute to advanced staging while controlling for potential confounding factors.

Regression models for advanced tumoral stages (III, IV) were built using backward elimination (*p* > 0.15) with an order of elimination based on clinical evaluation and p values. In addition to sex (male/female), and age (continuous variable, years), we considered a positive FIT (yes/no) and being a non-smoker (yes/no). 

## 3. Results

### 3.1. Patient’s Characteristics

Table 1 summarizes the baseline characteristics of our cohort. Our prospective registry comprised 245 patients, with 42% females and all patients diagnosed with CRC. The primary reasons for referral were a positive FIT (23.4%), anemia (18.7%), hematochezia (16.6%), abdominal pain (6%), changes in bowel habits (5.1%), and a combination of two or more of these symptoms (19.6%). The prevalent comorbidities among our patients included diabetes (27.3%), obesity (29.4%), and smoking (14.5%), with 26.1% identifying as ex-smokers.

The predominant method of referral was the electronic consultation system (36.7%), followed by the CRC screening program (14.8%) and direct referrals from family physicians (11%). Among the patients, 42% were referred to our endoscopy unit before 2019 and 2020, while 58.1% were sent to us in 2021 and 2022 (Table 2).

The most frequent tumor location was the right colon (34.6%), followed by the descendant and sigmoid (30.6%), rectum (29%), and transverse (3.8%). In terms of staging, 4.6% of patients had stage 0 tumors, 25.3% had stage I, 42.3% had stage II, 5.2% had stage III and 22.7% had stage IV.

### 3.2. Electronic Consultation Impact

The primary impact of electronic consultation as the main referral method for our patients was a notable reduction in diagnosis delay. Following its implementation in April 2020, a significant improvement in diagnosis times was evident (26 ± 10 days vs. 68 ± 50 days; *p* < 0.0001). After the full implementation of the eConsult, more women were diagnosed, with less cases from the screening program and also a lower proportion of patients with a positive FIT were seen among CRCs (Table 2). Notably, a higher proportion of patients from the eConsult presented with symptoms compared to other referral origins, where asymptomatic FIT-positive patients were more prevalent (85.2% vs. 73.3%; *p* = 0.033). However, no significant differences in CRC staging were observed between patients from the eConsult and those referred by other routes (Table 3). 

### 3.3. Colorectal Cancer Screening

Patients enrolled in the CRC screening program exhibited a higher prevalence of stage 0–II cancers compared to those presenting with symptoms (42.4% vs. 23.4%; *p* = 0.022). Furthermore, this subgroup was notably younger (63 ± 7 years vs. 68 ± 11 years; *p* < 0.0001), and there were no statistically significant differences in waiting times for colonoscopy (42.6 vs. 48.2 days). 

### 3.4. Factors Related to Tumoral Staging

We observed no differences in the distribution of early (stages 0–II) versus advanced (III–IV) cancers when considering the year of diagnosis (before or after May 2020) or referral pathway (eConsult vs. traditional referrals). However, a significantly higher proportion of early-stage cancer (0–II) was identified in patients from the CRC screening program compared to those with symptoms. This difference was also evident in patients referred for colonoscopy due to a positive FIT outside the CRC screening program (36.5% vs. 16.3%; *p* = 0.002). Of note, we observed a higher proportion of stage IV tumors in the right colon compared to the rest of the stages (44.2% vs. 29.9%; *p* = 0.029) and a lower proportion of stage IV in the sigmoid colon (18.6% vs. 35.7%; *p* = 0.03). There were no significant differences regarding tumoral stages in the rest of the colon segments. In this sense, while only 9.3% of patients referred because of a positive FIT had stage IV CRCs, 25.6% of the patients with other symptoms had *p* = 0.014. We did not find any differences the in tumoral stages related to obesity or diabetes. 

Examining the delay for colonoscopy, we noted that a higher proportion of advanced cancers were diagnosed within 30 days (64% vs. 40%; *p* = 0.003). Notably, the advanced-stage group exhibited a higher rate of active smokers (15.2% vs. 11.5%, *p* < 0.0001), while the proportion of patients who had never smoked was higher in the early-stage group (64.8% vs. 40.4%; *p* = 0.002). Table 3 illustrates the differences between early and advanced stages.

Multivariate logistic regression, as shown in Table 4, indicated that FIT referrals and being a never-smoker were identified as protective factors against advanced staging.

## 4. Discussion

Our study highlights the impact of eConsult in reducing diagnosis delay in CRC without compromising the staging outcomes. Furthermore, our results underscore the efficacy of FIT and screening programs in identifying colorectal cancers at earlier stages, particularly among a relatively younger cohort, and highlight the efficiency in the timely scheduling of diagnostic procedures.

Access to specialty care poses a potential barrier leading to diagnosis delays and adverse patient outcomes [14]. The implementation of eConsult between GPs and specialists has demonstrated its efficacy in prioritizing patients with urgent health concerns, improving waiting times, and addressing GP requests without unnecessary face-to-face consultations [15]. While our study focused on reducing diagnosis delay, it is worth noting that telemedicine experiences, such as eConsult, can have other broader economic, psychological, comfort, and environmental consequences, aspects that were not specifically measured but have been explored in previous studies [12]. The economic impact has been extensively studied, given the interest it arouses in healthcare systems, both public and private. For example, Wanigasooriya and colleagues analyzed 1531 patients comparing the outcomes of virtual clinics (VC) in 2020 with face-to-face (FtF) clinics in 2019. VCs saved GBP 7482.97 over five months, with estimated annual savings of GBP 17,959.13 for patients and GBP 192,580.80 for the NHS. Furthermore, VCs saved 9288 travel miles, reducing CO_2_ emissions by 0.7 metric tonnes [12]. Patient satisfaction with eConsults is generally high, according to studies measuring it through surveys [16]. Benefits include faster access to care and avoiding face-to-face referrals. However, qualitative studies reveal a trade-off: while patients appreciate the speed and convenience, they miss the opportunity for in-person conversations with specialists about treatment preferences. Exclusion from clinical conversations has implications, with patients desiring transparency and assurance about comprehensive communication between primary care providers (PCPs) and specialists. 

During the lockdown, between March and June 2020, referrals for colorectal cancer suspicion from primary care to the endoscopy units were significantly challenged, with a likely impact on the patients’ survival, since diagnostic and therapeutic delays might presumably have an influence on mortality [17,18,19]. In CRC referrals, according to some data from Spanish primary care centers via the traditional (paper based) referral pathway, the mean diagnostic delay from patients’ first contact with the family physician can take up to 124 days [20], despite the government’s rule of no more than 30-day delays for endoscopic diagnostic procedures. Shortly after lockdown, the Spanish Society of Gastrointestinal Endoscopy launched a consensus guideline for the prioritization of endoscopic procedures in a reduced endoscopic practice [13]. Regarding colorectal cancer, referrals for CRC symptoms or screening were considered a priority. Our study shows that an earlier diagnosis of CRC, within almost 30 days, was made for patients in the eConsult era, especially in symptomatic patients, minimizing the patient’s anxiety and probably improving their perception of the quality of care. Although we did not observe earlier stages in the eConsult cohort, we still lack information about 5-year survival rates. Previous studies have reported that diagnosis delays among symptomatic patients were not related to tumoral stage [21]; however, future studies with larger sample sizes and longer follow-ups should be performed to accurately assess the impact of diagnosis delay in tumor stage and survival. As expected, more CRC patients from eConsult had symptoms that triggered referrals. Symptoms, mostly hematochezia, altered bowel habits or abdominal pain normally appear during the more advanced stages of CRC, being the main complaint of patients who seek GP advice [22]. The main reason for this finding is that eConsult reduced diagnostic delays, mainly for symptomatic, and thus more advanced, CRC patients. Nevertheless, we observed a substantially diminished clinical and endoscopic referral demand throughout the six months that followed the lockdown enforced by the government that could have determined the bias toward higher CRC stages in the succeeding months. This effect could have been progressively lessened and prolonged, so we might see a trend towards earlier diagnoses in the eConsult patients in the coming years. Interestingly, eConsult also increased the rate of females diagnosed with CRC (Table 2), which also were overrepresented in advanced stages (Table 3), although this difference did not reach statistical significance. As a speculative hypothesis, we might state that women were more reluctant to clinical requests throughout the lockdown, and for this reason, more advanced neoplasms might have been found among them.

As the CRC screening program was quickly resumed, waiting times did not change and, as expected, patients had a higher rate of early-stage cancer, a fact previously described and reported in the main CRC screening program, in asymptomatic referrals with a positive FIT [23]. This is especially relevant in a situation in which, for patient-related or healthcare-system-related reasons, less eligible individuals underwent CRC screening worldwide [24]. Indeed, the only factor directly related to an earlier stage at diagnosis was the absence of symptoms and a FIT positive referral, either inside the CRC screening program or as part of this opportunistic screening performed by GPs in patients not compliant with the screening program. 

Our study has some limitations. First, being a single-center study, its generalizability may be limited, but it provides insights into the communication systems and barriers between GPs and specialists in clinical practice. Secondly, the relatively short follow-up period limits the information about survival, although extrapolation can be made based on the stage at diagnosis. Third, the absence of a control group introduces the possibility of missing external factors that could influence the results. However, it accurately reflects real-world practices in CRC diagnosis. Lastly, since it was performed during the COVID-19 pandemic; therefore, the results may be subject to the overestimation of delays during this period. Nonetheless, data post-eConsult implementation indicate lower delays in diagnosis compared to other studies previously mentioned [20].

## 5. Conclusions

In conclusion, our study underscores the independent protective effect of a positive FIT test with no symptoms as the main referral cause against advanced CRC. In addition, eConsult significantly reduces diagnostic delay without a direct impact on staging. Further research with extended follow-up periods is essential to explore the long-term implications of these findings. Current evidence for important outcomes of e-consultation is generally positive, but the overall quality of evidence is generally low, since most studies are observational, reinforcing the need for further studies.

## Figures and Tables

**Table 1:** Patients’ general characteristics.

Parameter	n	%
**Age** (median, years)	67 ± 11	
**Diagnosis delay** (days)	43 ± 77	
**Sex**		
Male	138	58
Female	100	42
**Comorbidities**		
Active smoker	34	14.5
Ex-smoker	62	26.1
Never-smoker	136	55.5
Diabetes	67	27.3
Obesity	70	29.4
**Referral reasons/Indication**		
FIT positive	55	23.4
Anemia	44	18.7
Hematochezia	39	16.6
Change in bowel habits	12	5.1
Abdominal pain	14	6
Constitutional syndrome	8	3.4
Family history	1	0.4
More than one	46	19.6
Other	16	6.8
**Referral precedence**		
CRC screening program	53	14.8
Primary care	26	11
eConsult	89	36.7
Gastroenterology dept	23	9.7
Inpatients	4	1.7
Emergency	33	13.9
Other	27	11.4
**Year**		
2019	11	4.7
2020	88	37.3
2021	122	51.7
2022	15	6.4
**Tumor location**		
Right colon	82	34.6
Transverse	9	3.8
Descendant-sigmoid	75	30.6
Rectum	71	29
**Tumor staging**		
Stage 0	9	4.6
Stage 1	49	25.3
Stage 2	82	42.3
Stage 3	10	5.2
Stage 4	44	22.7

**Table 2:** Differences between pre and post-eConsult full implementation.

	Pre-eConsult	Post-eConsult	*p*
Sex (female) (%)	30%	44.1%	0.002
Procedence (eConsult ^◆^) (%)	16%	73.2%	<0.0001
Screening program (%)	22.2%	9.6%	0.007
Positive FIT * (%)	23.2%	21.8%	0.79
Obesity (%)	36.4%	23.5%	0.032
Delay > 30 days (%)	53.5%	31.1%	0.001
Tumoral stage (III–IV) (%)	70%	76%	0.4
Location (right colon) (%)	41%	36%	0.72
Location (rectum)(%)	31%	26%	0.27
Age (years; mean ± SD)	66 ± 10	68 ± 11	0.121
Diagnostic delay (days; mean ± SD)	68 ± 50	26 ± 10	<0.0001

^◆^ Electronic consultation; * fecal immunological test.

**Table 3:** Factors influencing tumor staging.

	Stage 0–II	Stage III–IV	*p*
Year (2021–2022) (%)	46.2%	53.4%	0.37
Procedence (eConsult ^◆^) (%)	35.8%	34%	0.81
Screening program (%)	26%	12%	0.023
Positive FIT * (%)	36.5%	16.3%	0.002
Sex (female) (%)	30.2%	41.9%	0.28
Obesity (%)	32.1%	31.1%	0.89
Active smoker or ex-smoker (%)	57.7%	35%	0.002
Age (years; mean ± SD)	65 ± 8	68 ± 11	0.18
Diagnostic delay (days; mean ± SD)	57 ± 13	42 ± 22	0.25

^◆^ Electronic consultation; * fecal immunological test.

**Table 4:** Multivariate analysis for advanced CRC (stages III and IV).

	Hazard Ratio	95% CI	*p*
Sex	0.99	0.53–1.89	0.99
FIT * vs. Other	0.39	0.18–0.87	0.02
Non-smokers	0.25	0.11–0.55	0.001
Age	1.01	0.98–1.05	0.57
Location (right vs. left)	1.13	0.54–2.44	0.74
Diagnosis delay (days)	0.99	0.99–1	0.18

* Fecal immunological test.

## Data Availability

The data presented in this study are available upon request from the corresponding author. The data are not publicly available due to ethical concerns.
